# English phonology and an acoustic language universal

**DOI:** 10.1038/srep46049

**Published:** 2017-04-11

**Authors:** Yoshitaka Nakajima, Kazuo Ueda, Shota Fujimaru, Hirotoshi Motomura, Yuki Ohsaka

**Affiliations:** 1 Kyushu University, Department of Human Science/Research Center for Applied Perceptual Science, Fukuoka, 815-8540, Japan; 2 Kyushu University, Graduate School of Design, Human Science Course, Fukuoka, 815-8540, Japan; 3 Kyushu University, The 21^st^ Century Program, Fukuoka, 819-0395, Japan

## Abstract

Acoustic analyses of eight different languages/dialects had revealed a language universal: Three spectral factors consistently appeared in analyses of power fluctuations of spoken sentences divided by critical-band filters into narrow frequency bands. Examining linguistic implications of these factors seems important to understand how speech sounds carry linguistic information. Here we show the three general categories of the English phonemes, i.e., vowels, sonorant consonants, and obstruents, to be discriminable in the Cartesian space constructed by these factors: A factor related to frequency components above 3,300 Hz was associated only with obstruents (e.g., /k/ or /z/), and another factor related to frequency components around 1,100 Hz only with vowels (e.g., /a/ or /i/) and sonorant consonants (e.g., /w/, /r/, or /m/). The latter factor highly correlated with the hypothetical concept of *sonority* or *aperture* in phonology. These factors turned out to connect the linguistic and acoustic aspects of speech sounds systematically.

The concept of the syllable^1^ is important to understand how speech phonemes are connected with one another in time. However, there are hardly any acoustically-based investigations of phonemes from such a viewpoint. We were particularly interested in whether the three-factor spectral representation of speech sounds reported by Ueda and Nakajima^2^ could be related to phonological categories such as vowels and consonants, or as sonorants and obstruents^1^. Ueda and Nakajima analysed critical-band-filtered power fluctuations of speech signals in eight different spoken languages/dialects, and obtained three factors common to all of these languages/dialects. Two of these factors each had one prominent peak area in factor loadings plotted as functions of frequency, and the remaining factor exhibited two prominent peak areas. The crossings of these factor-loading curves separated four frequency bands that were similar across these languages/dialects. These four bands were used by our research group to generate noise-vocoded speech in Japanese and German when the present analysis was on the way, and the generated signals indicated high intelligibility of up to 95%^3^. These results were consistent with representative past data on noise-vocoded speech^4^,^5^.

This led to the idea that the three factors yielding these four frequency bands might be closely related to syllabic structures of speech. Fortunately, a speech database of British English^6^ was available for examining this hypothesis, and thus we checked the correspondence between the factor scores and the phonemic labels. British English would give us a reliable starting point, because its phonology has been described thoroughly in the literature^1^,^7^,^8^.

## Results

We analysed the spoken sentences with the aim of extracting the three factors^2^—they were designated as the *low & mid*-*high* factor, which appeared in two frequency ranges around 300 and around 2,200 Hz, the *mid*-*low* factor around 1,100 Hz, and the *high* factor above 3,300 Hz (Supplementary Fig. S1). For each phonemic period labelled in the database, the factor scores at the temporal midpoint were considered to be representative (as a first step of this exploration) (Supplementary Fig. S2).

**Figure 1:**
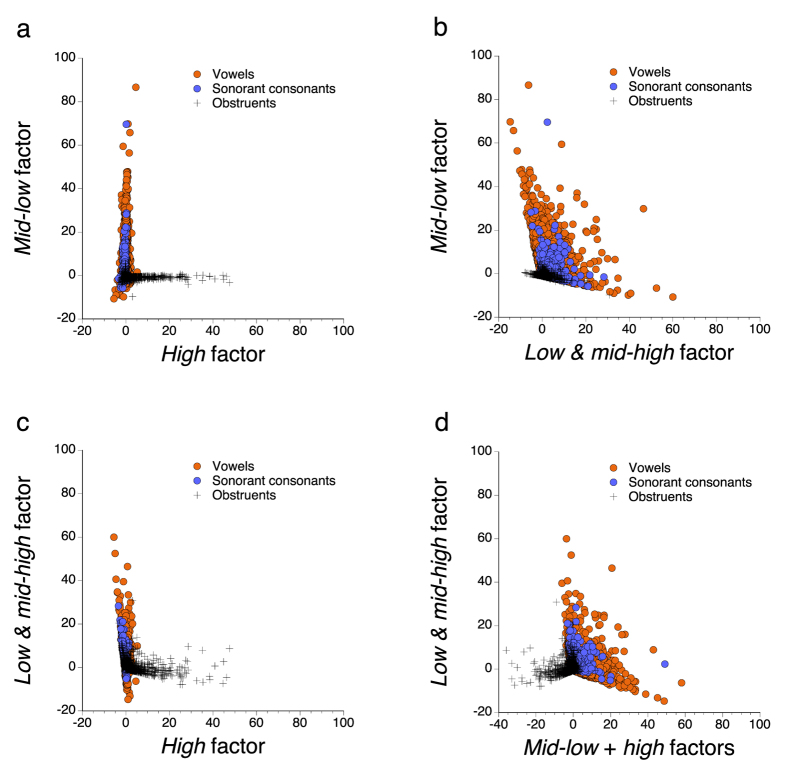
Distribution of uttered phonemes in the three-dimensional factor space. The three phonological categories (vowels, sonorant consonants, and obstruents) are differentiated. The panel (**d**) shows how the three-dimensional configuration looks if viewed from above-right in the panel (**a**); the horizontal axis is derived from the combination of the *mid*-*low* factor and the *high* factor, calculating 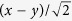, where *x* signifies the coordinate of the *mid*-*low* factor, and *y* that of the *high* factor.

Each labelled phoneme period was represented as a point in the three-dimensional Cartesian space of which the three factor scores comprised the coordinates. The distribution of uttered phonemes in this factor-score space showed an unexpectedly characteristic shape. The distribution observed in the plane (two-dimensional space) of the *high* factor and the *mid*-*low* factor displayed an L-shaped pattern. The densest point was close to the origin, stretching two linear arms along both axes in the positive directions. In the three-dimensional space with the *low & mid*-*high* factor added, the L-shaped distribution was represented by two distinct walls connected in a right angle at the densest point, and tapering off with increasing distance from that point (Fig. 1d and Supplementary Figs 3–5).

**Figure 2:**
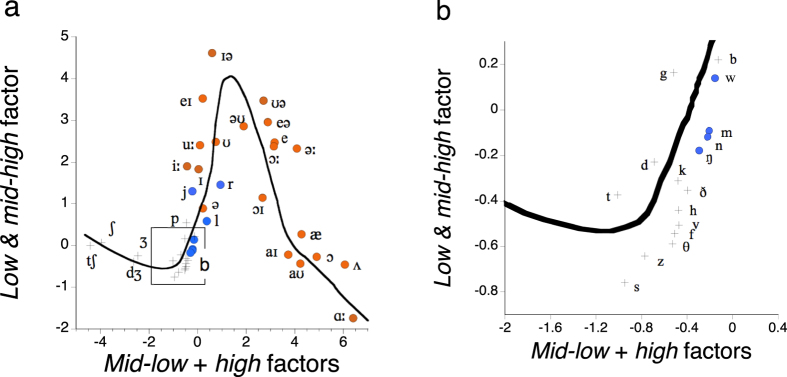
Configuration of British English phonemes obtained by averaging all uttered samples of each phoneme in the three-dimensional factor space. Each point indicated by an International Phonetic Alphabet symbol represents 27–2172 samples. The curve shows a fitting by eye of a sonority/aperture scale as in the linguistics literature^1^,^7^,^14^,^15^. The direction of this view is the same as in Fig. 1d.

The factor scores for each English phoneme were averaged across the three speakers (Supplementary Figs 6–8), and thus each English phoneme was represented by one point in the 3-dimensional factor space (Fig. 2).

## Discussion

*Vowels* and *obstruents* were separated very clearly, and *sonorant consonants* occupied an area in-between. Sonorant consonants sometimes play roles similar to those of vowels in the sense that some of them can be syllable nuclei in English. However, they can never be nuclei of stressed syllables. It is to be noted that the schwa /ə/, which can be a syllable nucleus but cannot be a nucleus of a stressed syllable, was located in the middle of the sonorant-consonant area in Fig. 2. Those arguments also held for the factor scores of individual speakers (Supplementary Figs S3–S8) with a few exceptions of stop consonants uttered by female speaker 2; her stop consonants were sometimes contaminated with clearly audible puffs on the microphone, and this very probably caused the exceptions. The factor space well reflected the phonological (linguistic) roles of the phonemes^1^,^7^.

These three factors should be involved in the perception of the phonemes, because they are directly connected to the functions of the auditory periphery associated with critical bands^9^,^10^,^11^,^12^,^13^. The configuration of the phonemes in Fig. 2 can be related to *sonority*, or *aperture* (Table 1), as defined in phonology^1^,^7^,^14^,^15^,^16^,^17^,^18^,^19^; vowels, sonorant consonants, and obstruents make a hierarchy of sonority in phonology^1^. The three categories of phonemes were located in the order of *sonority* in the map as indicated above. Sonority is a phonological concept created to describe the structures of syllables. It is considered that low vowels typically have high sonority and stop consonants low sonority, and ordinal scales of sonority has been proposed a few times in linguistics. One of the classic examples in this respect is the scale of *aperture* proposed by de Saussure^14^, and we will examine his classic scale as a potential tool to analyse English speech sounds quantitatively. A syllable of an English word can be described as a temporal contour of sonority that has a peak in its nucleus, which is usually a vowel. The contour typically rises monotonously before the peak, and declines monotonously after the peak. An illustrative example is the English word “trunk,” which is made of a single syllable with a clear single peak preceded by an ascending series of sonority and followed by a descending series. On the other hand, the word “apple” is considered to be made of two syllables although it has only one vowel. The reason is that, in the series of phonemes /æpl/, the /l/ is clearly higher in sonority than the preceding /p/, and that there should be a separate syllable here. If the same phonemes are rearranged in the order /ælp/ (alp) or /læp/ (lap), however, there is just one syllable with a peak at /æ/. The frequency zone below 3,000 Hz, in which the first three formants of vowels are located, is called the sonorant frequency zone^7^, and thus the concept of sonority has been closely related to the acoustic aspects of speech. However, it has never been related to real acoustic measurement systematically. A very recent attempt in linguistics is to quantify well-formed and ill-formed syllables on the basis of sonority^18^,^19^, and this attempt, if supported by the present acoustic analysis, is very likely to create a new area of linguistics.

A Spearman’s rank correlation coefficient was calculated between each obtained factor and each sonority/aperture scale from the linguistics literature^1^,^7^,^14^,^15^; factor scores were averaged for each English phoneme. All three factors extracted here—by a purely acoustic analysis based on critical bands^9^,^10^,^11^,^12^,^13^—had significant correlations with sonority/aperture (Table 2). The *low & mid*-*high* factor and the *mid*-*low* factor had positive correlations, and the *high* factor negative correlations. This could be associated with the fact that the three phonological categories, i.e., vowels, sonorant consonants, and obstruents, had clearly defined areas in the factor space as in Fig. 1. Since the *mid*-*low* factor always showed a high positive correlation, we may take up this factor as a first approximation of sonority. [Examples of vowels, sonorant consonants, and obstruents were extracted from the spoken sentence in Supplementary Fig. S2. They produced *mid*-*low* factor scores as indicated in parentheses below, which are considered a first approximation of sonority. An audio demonstration (Supplementary Audio S1) presents these sounds in the order from higher to lower mid-low factor scores: /ɔː/ (8.42), /aI/ (6.22), /ə/ (0.66), /r/ (0.04), /ŋ/ (−0.29), /d/ (−0.78), and /s/ (−0.73)].

Frequency components above 3,300 Hz, which had been excluded from the above-mentioned sonorant frequency zone^7^, are related to the *high* factor^2^, and this factor is negatively correlated with sonority/aperture. This means that these high-frequency components may *suppress* perceived sonority, but this possibility has never been explored in linguistics. Because human listeners have to extract linguistic information included in speech signals quickly and often in a noisy environment, it is very likely that the auditory system utilises such high components spreading over a broad frequency area to clarify syllable boundaries. This can be related to the controversial fact that the consonant /s/ has an exceptionally high probability to begin or end syllables in English^1^,^7^, compared to its position on sonority scales (Table 1). Frequency components above 3,300 Hz dominate in this phoneme. We can hypothesise that frequency components below 3,300 Hz raise, and that components above 3,300 Hz suppress sonority. The present study thus showed a necessity to understand syllable structures of British English by analysing recorded speech psychoacoustically. Specifically, the linguistic concept of *sonority* should be established in an acoustic framework, connecting linguistics and acoustics.

## Methods

### Speech samples

A database of British English speech was used (ATR British English Database^6^), in which all phoneme labels were linked to specific periods of speech signals. All the labelled phonemes sounded approximately as indicated if played separately. Two female and one male speakers uttered the same 200 sentences in this database. (The database included samples uttered by another male speaker, but the labelling data for this speaker were broken. We asked the company that issued the database to fix the problem, but this turned out impossible).

The labelling data of the database were modified in the following manner, because our direct purpose was to relate the present analysis to classic literature in phonology.

1. Closure periods of stop consonants were labelled as such in the original database. Those periods were omitted from the present analysis, because there was almost no sound energy in these periods, i.e., these periods were unobservable. This simplification was necessary as this was an exploratory attempt to connect the acoustic and phonological feastures of speech sounds.
2. If there was more than one label attached to a single linguistic phoneme, one representative label was chosen. Shorter periods and transient periods were not chosen.
3. If a label was different from any possible phonemes that appeared in dictionaries, the sound was omitted from analysis. Voiced-unvoiced mismatches between the dictionaries and the database were permitted, however.
4. Triphthongs were regarded as diphthongs obtained by omitting the last portions. This modification was necessary because the database did not provide labels for triphthongs.

In the database, 31,663 phonemic periods were labelled, and 7,523 were omitted from further analyses.

### Factor analysis of power fluctuations of critical-band-filtered speech

All of the speech samples described in the previous section were jointly analysed as in Ueda and Nakajima^2^. The power fluctuations derived from the 20 critical-band filters^9^ were submitted to a principal component analysis in which three principal components were extracted, and a varimax rotation led to three factors that were to be related to four frequency ranges. Two different filter banks, A and B as in Ueda and Nakajima^2^, covering similar frequency ranges were used in order to check the stability of the analysis. The cumulative contributions for filter banks A and B, respectively, were 41 and 39% in the analysis of all the speakers, 41 and 39% in female speaker 1, 44 and 42% in female speaker 2, and 42 and 41% in male speaker 1. The following three factors appeared: *high* factor above 3,300 Hz, *mid*-*low* factor around 1,100 Hz, and *low & mid*-*high* factor in two frequency ranges around 300 and 2,200 Hz (Supplementary Fig. S1). This agreed with the results of Ueda and Nakajima^2^. The factor scores of these factors were expressed as functions of time, and, thus, three factor scores were given to each temporal point (Supplementary Fig. S2).

As a first step to relate this acoustic analysis to phonological aspects of the lexical phonemes, the position of each labelled acoustic sample was determined in the Cartesian space constructed by the three factor scores (Supplementary Figs S3–S5); the utterances of the three speakers were combined (Fig. 1). Since the results from filter banks A and B were very similar, only those from A were used. Each labelled acoustic sample was represented by its temporal centre portion; spectral fluctuation within each labelled period could include potentially important information, but such information was not utilised in the present analysis.

### Sonority scales and the phonemes of British English

We took up four cases in the linguistics literature (Table 1) in which sonority, or aperture, is defined systematically with an ordinal scale to classify phonemes^1^,^7^,^14^,^15^. Spencer’s scale is probably the most important because it covers all the English sounds with a minimum risk of confusion. We adjusted the proposed scales in order to apply them to the phonemes of British English:

1. English phonemes were first classified following the original authors’ explanations and examples as closely as possible.
2. Phonemes that could not be classified clearly were omitted from the analyses. Diphthongs and schwa were not included except for Spencer’s classification, in which there was a category of *vowels* in general.
3. In Harris’s scale, vowels and glides were classified into two categories, low vowels and high vowels/glides. We separated vowels according to the classification by Ladefoged^8^. Ladefoged classified vowels into four categories: high, mid-high, mid-low, and low. We regarded high and mid-high vowels as high vowels, and mid-low and low vowels as low vowels.

## Additional Information

**How to cite this article:** Nakajima, Y. *et al*. English phonology and an acoustic language universal. *Sci. Rep.* **7**, 46049; doi: 10.1038/srep46049 (2017).

**Publisher's note:** Springer Nature remains neutral with regard to jurisdictional claims in published maps and institutional affiliations.

## Supplementary Material

Supplementary Information

Supplementary Audio S1

## Figures and Tables

**Table 1:** Previously proposed sonority (aperture) scales.

Sonority (aperture) scale	Proposed category	Corresponding English phonemes
**de Saussure (1916/1959)^14^**		
6	a	/æ, αː,Λ
5	e, o, ö	/e, ɔ, ɔː/
4	i, u, ü	/I, iː, ℧, uː, j, w/
3	Liquids	/l, r/
2	Nasals	/m, n, ŋ/
1	Fricatives	/θ, ð, f, v, s, z, ʃ, ʒ/
0	Occlusives	/p, t, k, b, d, g/
**Selkirk (1984)^15^**		
10	a	/æ, αː, Λ/
9	e, o	/e, ɔ, ɔː/
8	i, u	/I, iː,℧,uː/
7	r	/r/
6	l	/l/
5	m, n	/m, n/
4	s	/s/
3	v, z, ð	/v, z, ð/
2	f, θ	/f, θ/
1	b, d, g	/b, d, g/
0.5	p, t, k	/p, t, k/
**Harris (1994)^7^**		
5	Low vowels	/æ, αː Λ, e, ɔ, ɔː/
4	High vowels and glides	/I, iː, ℧, uː, j, w/
3	Liquids	/l, r/
2	Nasals	/m, n, ŋ/
1	Fricatives	/θ, ð, f, v, s, z, ʃ, ʒ, h/
0	Plosives	/p, t, k, b, d, g/
**Spencer (1996)^1^**		
5	Vowels	/æ, αː, Λ, e, ɔ, %ː, ə, əː, I, iː, ℧, uː, aI, a℧, eə, eI, ɔI, ə℧, Iə, ℧ə/
4	Glides	/j, w/
3	Liquids	/l, r/
2	Nasals	/m, n, ŋ/
1	Fricatives and affricates	/θ, ð, f, v, s, z, ʃ, ʒ, h, tʃ, dʒ/
0	Plosives	/p, t, k, b, d, g/

**Table 2:** Spearman’s rank-order correlation coefficients between the sonority/aperture proposed in the linguistics literature^1^,^7^,^14^,^15^ and the factor scores obtained in the present analysis, averaged over the same phonemes.

Sonority/aperture scale	Factors		
	*Low & mid*-*high*	*Mid*-*low*	*High*
de Saussure (1916/1959)^14^	0.3415	0.8251*	−0.3597*
Selkirk (1984)^15^	0.3025	0.8708*	−0.2840
Harris (1994)^7^	0.3691*	0.8218*	−0.3863*
Spencer (1996)^1^	0.5380*	0.8347*	−0.4549*

Asterisks represent statistically significant correlation (*p* < 0.05).
