# Supplementary material for: English phonology and an acoustic language universal
Source: Sci Rep. 2017 Apr 11;7:46049. doi: 10.1038/srep46049 (PMC5387398; doi:10.1038/srep46049)
Supplement: Supplementary Information [file srep46049-s1.pdf]

Supplementary Information
English phonology and an acoustic language universal

Yoshitaka Nakajima[1*], Kazuo Ueda[1*], Shota Fujimaru[2], Hirotoshi Motomura[2], & Yuki Ohsaka[3†]

[1]Kyushu University, Department of Human Science/Research Center for Applied Perceptual Science, Fukuoka, 815-8540, Japan
[2]Kyushu University, Graduate School of Design, Human Science Course, Fukuoka, 815-8540, Japan
[3]Kyushu University, The 21st Century Program, Fukuoka, 819-0395, Japan
[†]Present address: Columbia University, School of Social Work, New York, NY 10027, USA
email to Y.N.: nakajima@design.kyushu-u.ac.jp; *email to K.U.: ueda@design.kyushu-u.ac.jp

Table of Contents

Supplementary Figures S1–S8
Supplementary Table S1
Supplementary Audio S1
References

Supplementary Figures

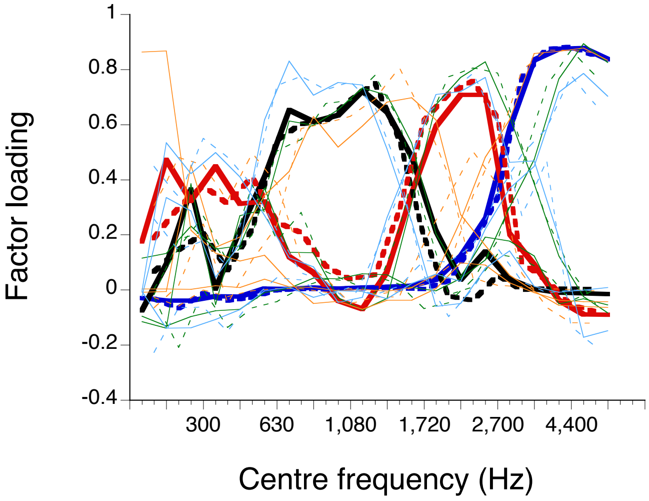

Supplementary Figure S1. Factor loadings plotted against the centre frequency of critical bands. The thick lines represent factor loadings derived from the merged data across the three speakers; the colours of the thick lines are to distinguish factors—the *low & mid-high* factor in red, *mid-low* factor in black, and *high* factor in blue. The thin lines show the results of the individual speakers without distinguishing factors within each speaker. The broken lines are the counterparts of the solid lines of the same colours, using a filter-bank shifted up by half a critical bandwidth (see Methods). The cumulative contributions were ranged from 39-44%. The same three factors as in Ueda and Nakajima[1] appeared in the present study.

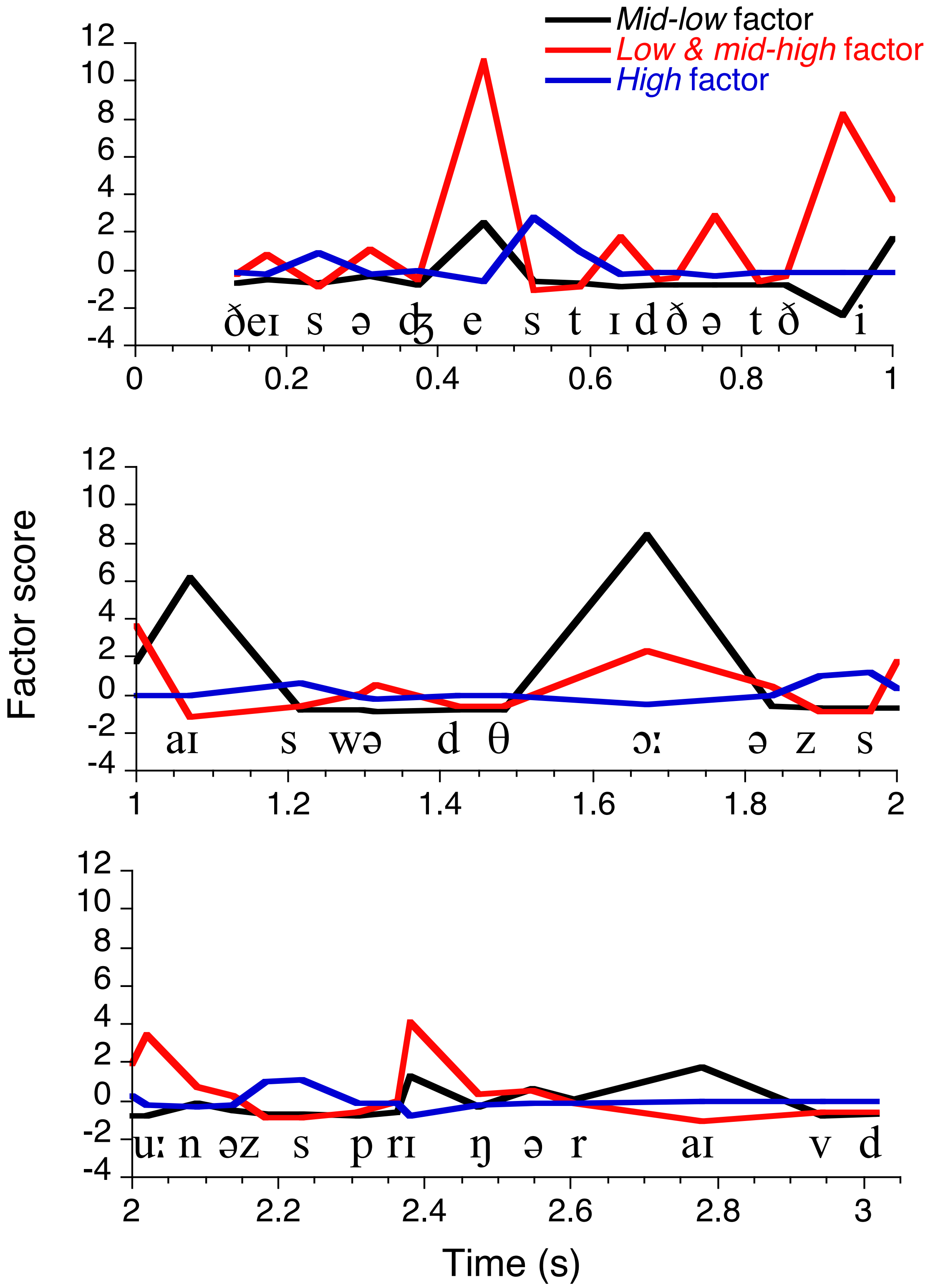

Supplementary Figure S2. An example of traces of factor scores along time axis. The sample sentence in Table S1 was uttered by a male speaker. The factor score for each phoneme at its middle point in time was indicated with an IPA label.

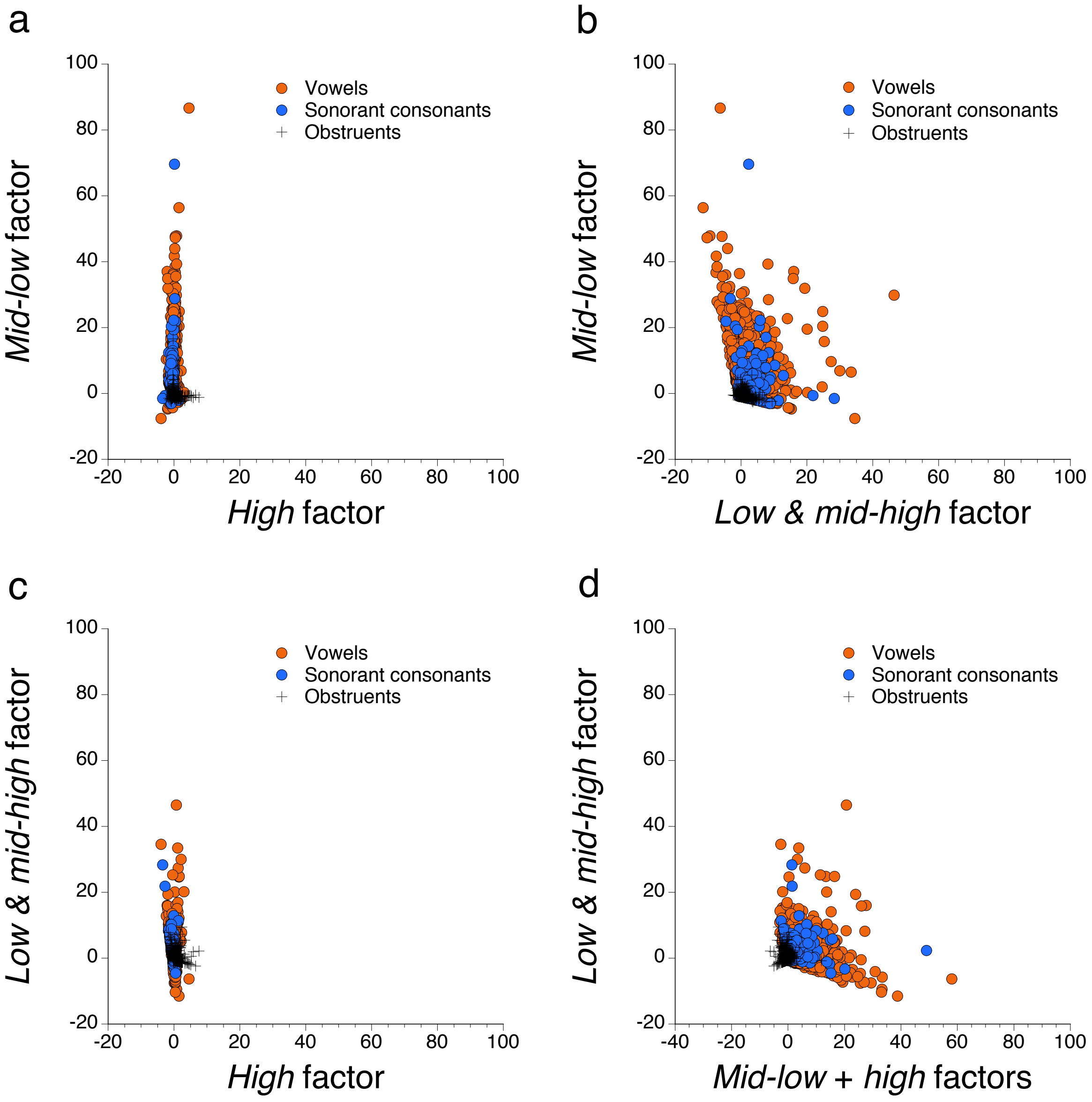

Supplementary Figure S3. Distribution of uttered phonemes by female speaker 1 in the three-dimensional factor space. The three phonological categories (vowels, sonorant consonants, and obstruents) are differentiated. The panel (**d**) shows how the three-dimensional configuration looks if viewed from above-right in the panel (**a**); the horizontal axis is derived from the combination of the *mid-low* factor and *high* factor, calculating $(x-y)/\sqrt{2}$, where x signifies the coordinate of the *mid-low* factor, and y that of the *high* factor.

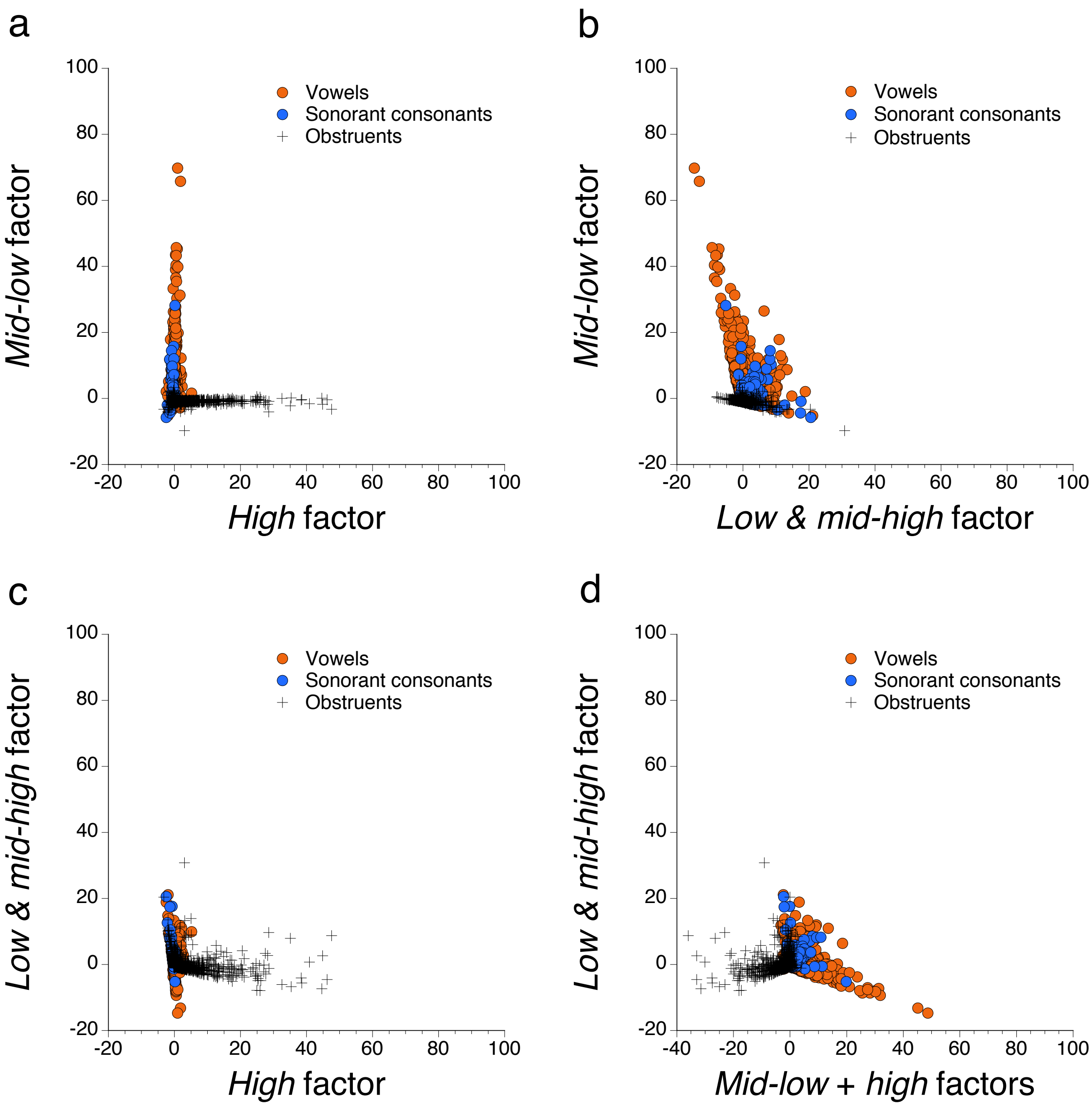

Supplementary Figure S4. Distribution of uttered phonemes by female speaker 2 in the three-dimensional factor space. See the caption of Supplementary Fig. 3 online for details.

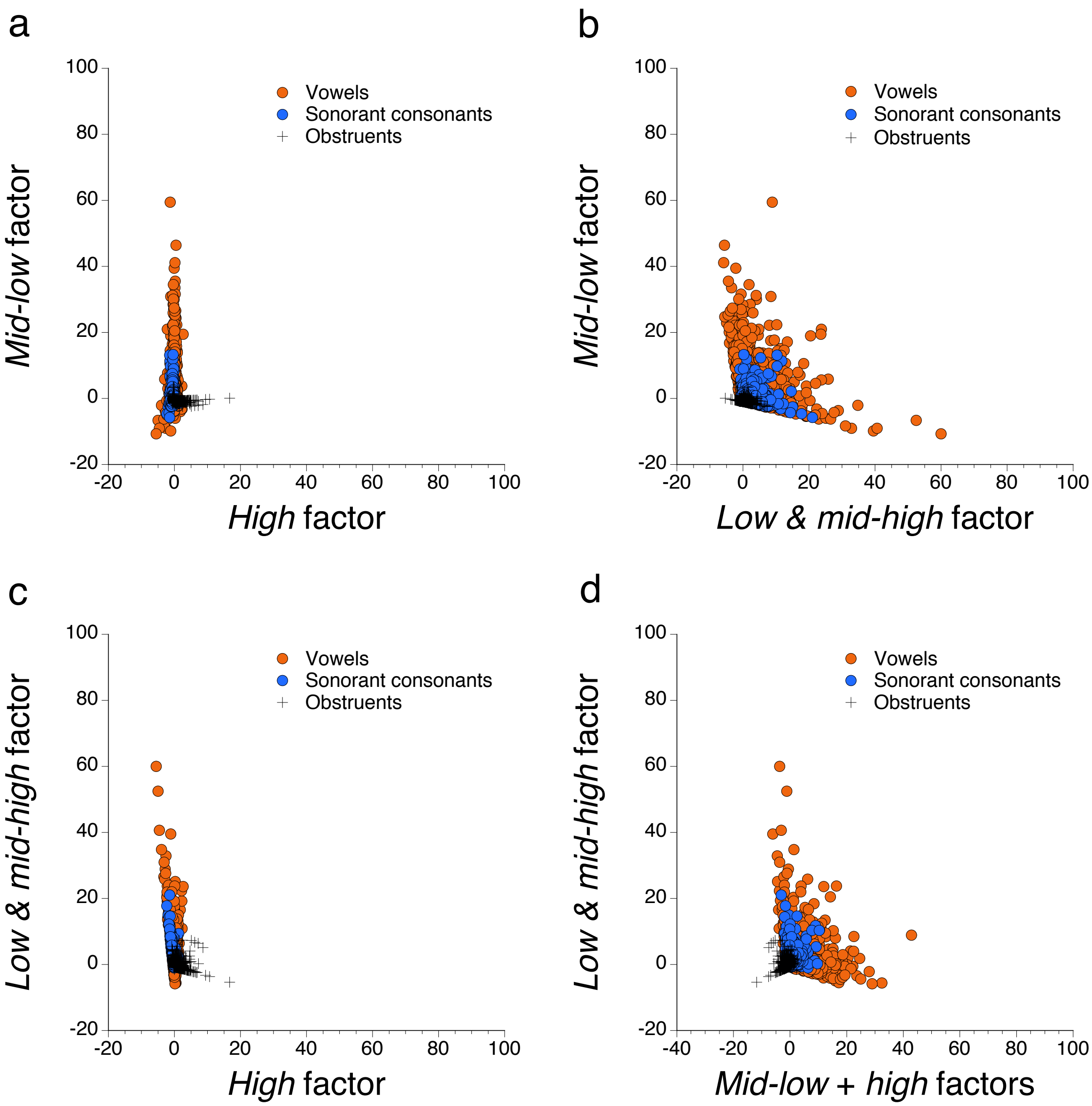

Supplementary Figure S5. Distribution of uttered phonemes by male speaker 1 in the three-dimensional factor space. See the caption of Supplementary Fig. 3 online for details.

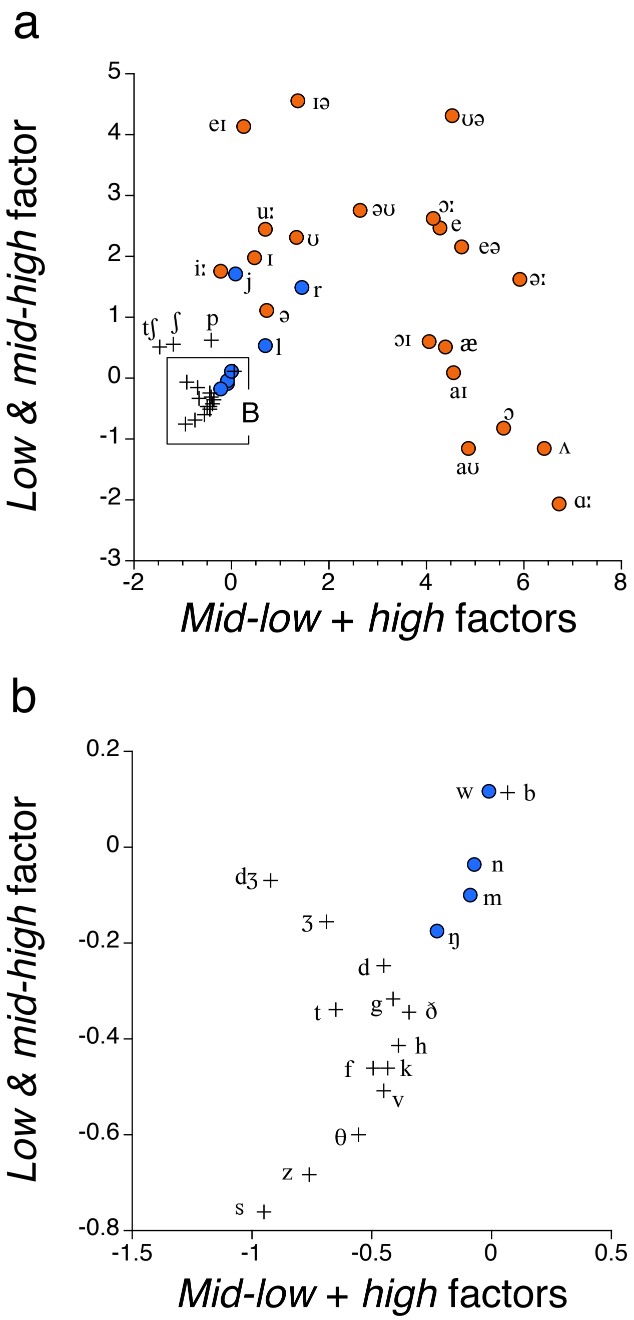

Supplementary Figure S6. Averaged configuration of British English phonemes uttered by the female speaker 1. The direction of this view is the same as in Fig. 1d.

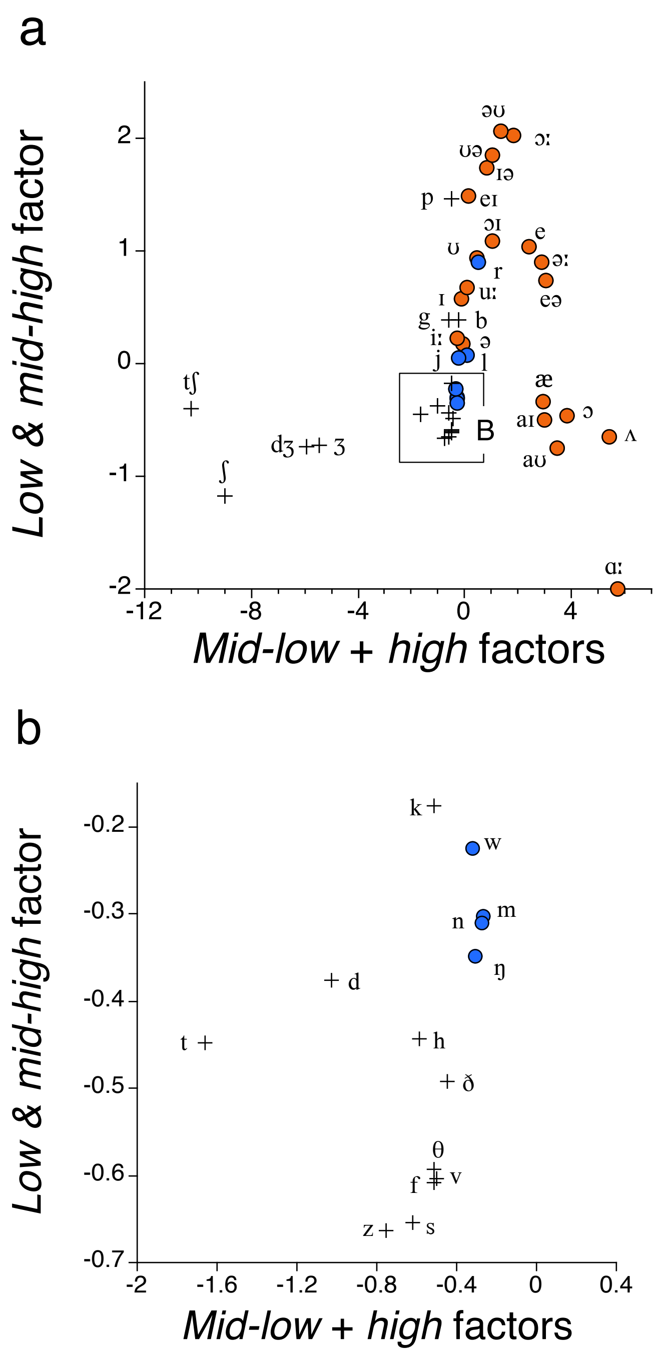

Supplementary Figure S7. Averaged configuration of British English phonemes uttered by the female speaker 2. The direction of this view is the same as in Fig. 1d.

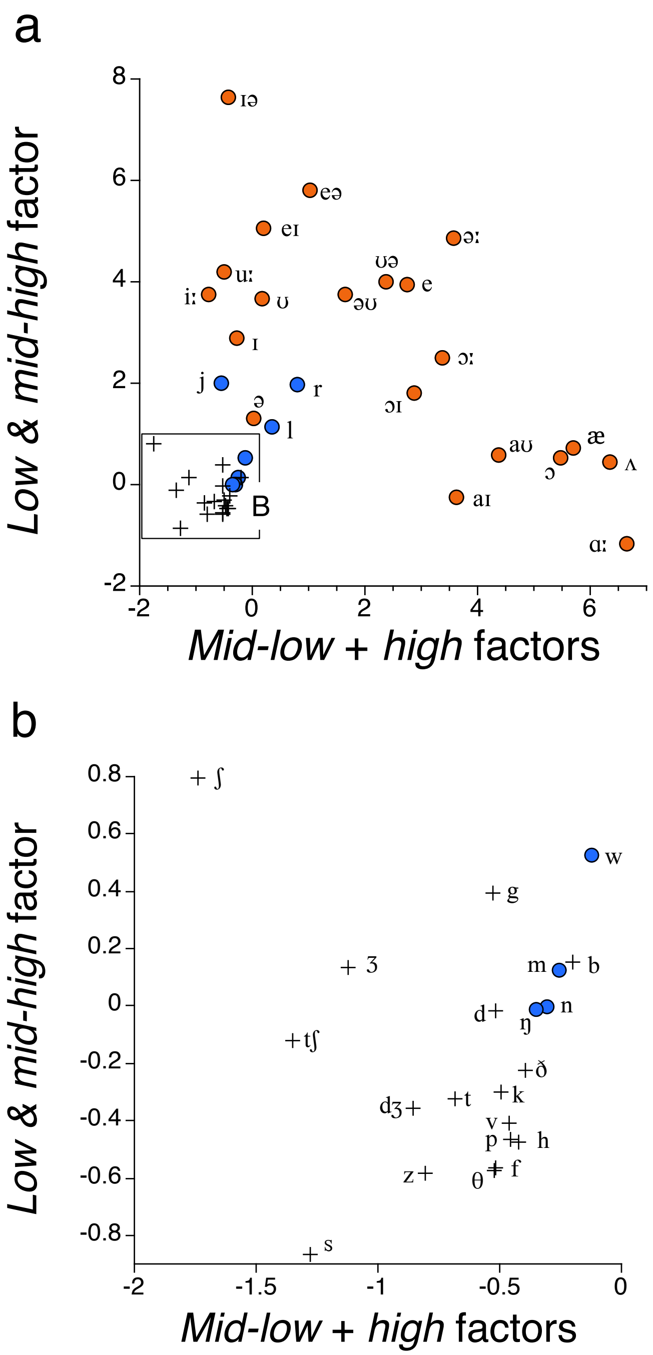

Supplementary Figure S8. Averaged configuration of British English phonemes uttered by the male speaker 1. The direction of this view is the same as in Fig. 1d.

Supplementary Table

Supplementary Table S1. A sample sentence's orthography and its corresponding representation in the International Phonetic Alphabet (IPA).

Type of representation	Samples											
Orthography	They	suggested	that	the	ice	would	thaw	as	soon	as	spring	arrived.
IPA	/ðeɪ	sədʒestɪd	ðət	ði	aɪs	wəd	θɔː	əz	suːn	əz	sprɪŋ	əraɪvd/

Supplementary Audio

Supplementary Audio S1. How the *mid-low* factor works as a first approximation of *sonority.* Excerpts from ATR British English Database[2] with permission.

References

1. Ueda, K. & Nakajima, Y. An acoustic key to eight languages/dialects: Factor analyses of critical-band-filtered speech. *Scientific Reports* (in press).
2. Campbell, N. The ATR British English speech database. Tech. Rep., ATR Interpreting Telephony Research Labs. (1993).
